# Supplementary material for: Human Periodontal Ligament Stem Cells (hPDLSCs) Spontaneously Differentiate into Myofibroblasts to Repair Diabetic Wounds
Source: Bioengineering (Basel). 2024 Jun 12;11(6):602. doi: 10.3390/bioengineering11060602 (PMC11200790; doi:10.3390/bioengineering11060602)
Supplement: Supplementary file 1 [file bioengineering-11-00602-s001.zip › bioengineering-2886035-supplementary.pdf]

Supplementary Materials

Human Periodontal Ligament Stem Cells (hPDLSCs) Spontaneously Differentiate into Myofibroblasts to Repair Diabetic Wounds

Yuxiao Li [1,2,3,†], Qi Su [1,2,3,†], Zhaoyu Tao [1,2,3], Xiang Cai [1], Yueping Zhao [2,3], Zhiying Zhou [2,3], Yadong Huang [1] and Qi Xiang [1,*]

3. Identification of periodontal stem cells

3.1. Flow cytometry

PDLSC was inoculated into 6-well plates and cultured at 37℃ and 5% CO2. After cell proliferation to 70% and fusion, discard the culture medium, wash the cells with sterile PBS twice, add 2mL trypsin solution with a concentration of 3mg/L for 1min, and neutralize with the culture medium. The neutralized cell suspension obtained was centrifuged at 2000 rpm for 5min, the supernatant was discarded, and the cells were re-suspended with PBS containing 2% FBS to obtain single-cell suspension of periodontal membrane cells. Antibodies CD44, CD90 and CD105 were added and incubated at 4℃ for 30 min. After washing with PBS, 500uL PBS cells containing 2% FBS were used for cytometry (CytomicsTMFC 500 MCL/MPL, Beckman Coulter, CA, USA), and the positive rate of each indicated antigen cell was calculated using cytometry software. It is expressed in percentage (%). (Figure S1).

3.2. Osteoblast and adipocyte differentiation

The 7th passage of PDLSCs was inoculated into 6-well plates, and L-DMEM medium containing 5% FBS was added, and cultured in an incubator at 37℃ and 5% CO2. After cell proliferation to 70% and fusion, the cells were divided into groups into osteogenesis (dexamethasone 100 n M, ascorbic acid 50 μg/ml, sodium β-glycerophosphate 5 mM; Sigma Corporation, AL, USA) and lipids (methyl isobutyl xanthine 0.5 mM, hydrocortisone 0.5 mM, indomethacin 60 mM; Sigma Corporation, AL, USA) induction medium was changed every 3 days. After 21 days of differentiation induction, each group was stained with alizarin red (Sigma Corporation, AL, USA) for bone differentiation and oil red O (Sigma Corporation, AL, USA) for lipid differentiation. (Figure S2)

Result

1. Isolation, purification and culture of PDLSCs

Primary cells were obtained by tissue block culture method. 1×10^3 periodontal stem cells of the second passage in good condition were inoculated in a 100 mm petri dish. After 7 days of culture, cells appeared around the tissue block and grew like clones at the bottom of the dish and radially around. 2 weeks later, the cells grew in a whirlpool shape under a light microscope, reaching about 80% fusion.

2. Evaluation of stem cell phenotypes

The isolated PDLSCs showed stem cell phenotype, and mesenchymal stem cell markers CD90, CD105 and CD44 were highly expressed on the surface of hPDLSCs (Figure S1).

3. Osteoblast and adipocyte differentiation

After 21 days of osteogenesis and lipogenesis, cells were stained with alizarin red and alcian blue, respectively. mineralized nodules can be formed 21 days after osteogenic induction, suggesting that PDLSCs have osteogenic differentiation potential (Figure 8A). 21d after adipogenic differentiation, the formation of dark blue lipid droplets can be seen by Alcian blue staining, suggesting that PDLSCs have the potential for adipogenic differentiation (Figure 8B). The calcification and lipid droplets in the osteogenic and lipogenic induction groups were clearly visible under the microscope, which confirmed that PDLSCs have the ability of Osteogenesis and adipogenic differentiation.

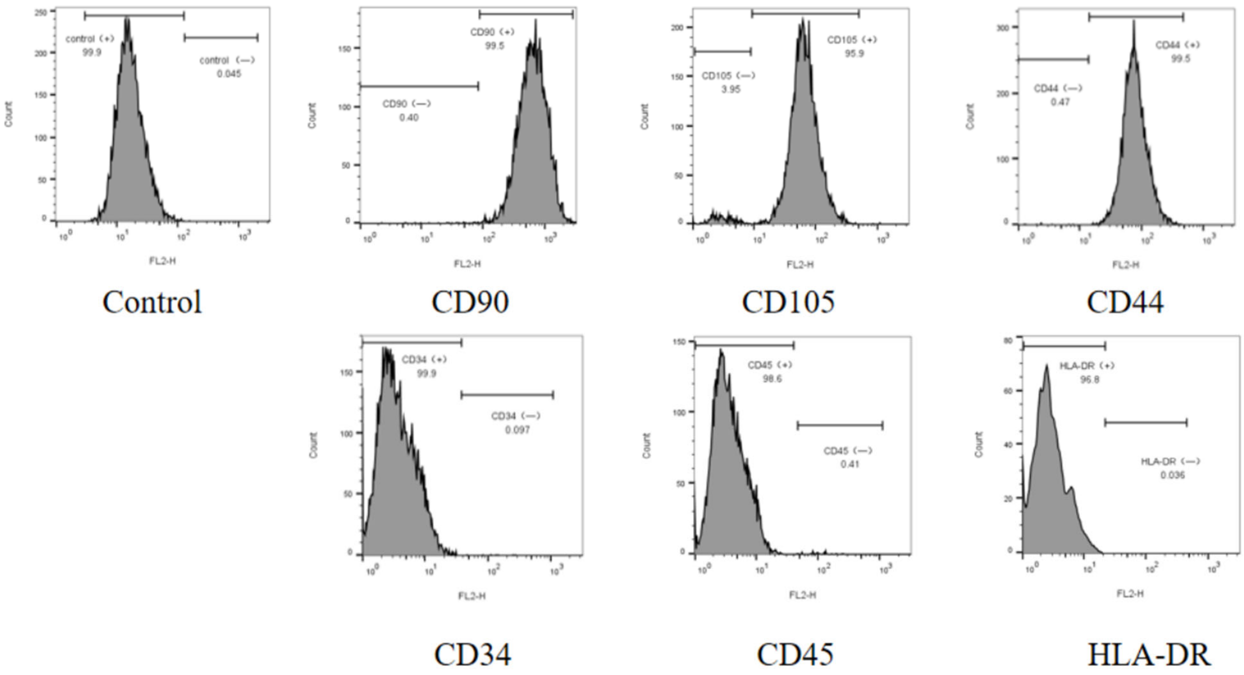

Figure S1. Surface markers of hPDLSCs mesenchymal stem cells were detected by flow cytometry.

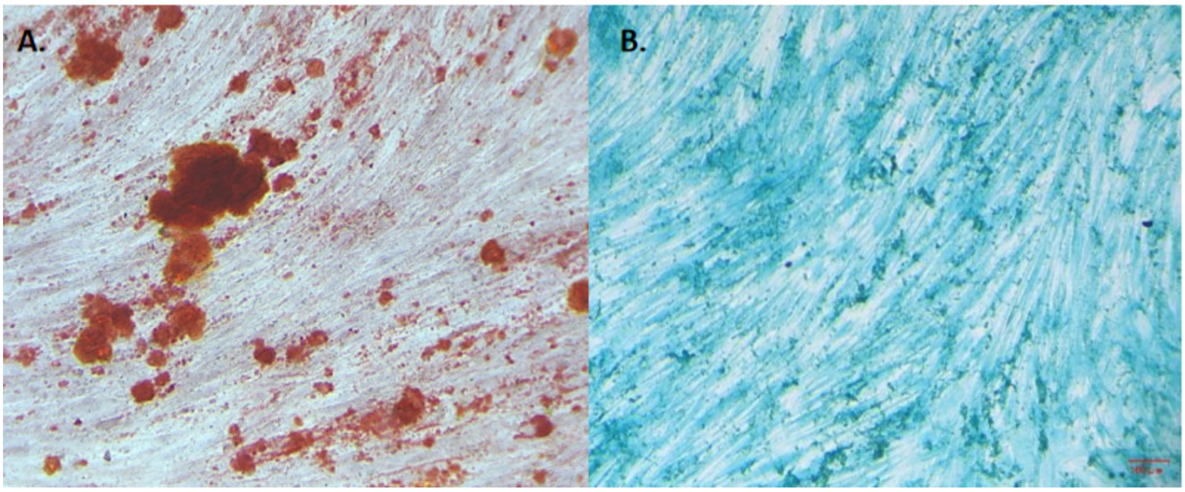

Figure S2. Differentiation potential of periodontal membrane stem cells. (**A**) In vitro osteogenic differentiation of periodontal membrane stem cells. (**B**) In vitro lipogenic differentiation of periodontal membrane stem cells.
